# Microbial respiration - a biomineral perspective

**DOI:** 10.1093/femsec/fiaf093

**Published:** 2025-09-25

**Authors:** Lucian C Staicu, Julie Cosmidis, Muammar Mansor, Catarina M Paquete, Andreas Kappler

**Affiliations:** Department of Bacterial Genetics, Faculty of Biology, University of Warsaw, 02-096 Warsaw, Poland; Department of Biological Sciences, Duquesne University, Pittsburgh, PA 15282, United States; Department of Earth Sciences, University of Oxford, OX1 3AN Oxford, United Kingdom; Department of Geosciences, University of Tübingen, 72070 Tübingen, Germany; Instituto de Tecnologia Química e Biológica António Xavier, Universidade Nova de Lisboa, 2780-157 Oeiras, Portugal; Department of Geosciences, University of Tübingen, 72070 Tübingen, Germany

**Keywords:** anaerobic respiration, biogenic particles, biomineralization, microbial biominerals, respiratory enzymes

## Abstract

Microbial biomineralization is a key process in natural and anthropogenic environments. Certain bacteria and archaea produce cellular energy via anaerobic respiration using metals and metalloids as terminal electron acceptors, producing intra- and extracellular biominerals. This article explores the biomineralization of arsenic (As), iron (Fe), sulfur (S) and selenium (Se), in relation with microbial respiratory processes. Ferric iron (Fe^III^) and the oxyanions of As, S and Se are used as terminal electron acceptors by specialized bacteria and archaea, providing significant amounts of energy under anoxic and nutrient-limiting conditions. These transformations result in the formation of various types of arsenic sulfides, iron (oxyhydr)oxides and sulfides, elemental S/S^0^ and elemental Se/Se^0^ biominerals, which will be the focus of this review. Certain biominerals (e.g. S^0^) function as storage compounds; others, like Se^0^, may increase the density and the buoyancy of bacteria harboring them or are by-products of this process. Arsenic sulfides and iron (oxyhydr)oxides and sulfides appear to be by-product biominerals or have a yet unknown function. The use of these biominerals as biosignatures is an open topic and an ongoing debate. Further exploration of the reviewed biominerals is needed from both fundamental and applied viewpoints, aspects which will be covered in this review.

## Teaser

Bacteria couple anaerobic respiration with biomineralization, generating biominerals of interest for fundamental and applied research.

## Introduction

Microbial biomineralization is the process by which microorganisms produce crystalline or amorphous minerals. This process can be genetically controlled and tightly regulated (e.g. magnetotactic bacteria produce intracellular magnetite and greigite minerals arranged in a linear structure–Uebe and Schüler 2021) or can occur as a by-product of microbial metabolism (extracellular iron minerals) (Staicu and Stolz 2021). From this perspective, two types of biomineralization can be identified: biologically controlled mineralization (BCM) and biologically induced mineralization (BIM) (Konhauser and Riding 2012). BCM implies a set of genes involved in the biomineralization, growth and localization of the biomineral, which is usually intracellular. BIM occurs as a result of the metabolic activity of the microbial cells which induce local chemical changes (e.g. pH rise), the minerals precipitating extracellularly (Cosmidis and Benzerara 2022). Some authors identify a third type, microbial influenced biomineralization, which, in fact, can be considered a subtype of BIM. This biomineralization entails that certain organic structures (e.g. Extracellular Polymeric Substances / EPS) act as nucleation surfaces for mineral precipitation in supersaturated solutions and it does not require cells to be alive or metabolically active (Cosmidis and Benzerara 2022). In contrast to BCM, in BIM and biologically influenced biomineralization there is no known genetic control on crystal nucleation or growth. Biomineralization in eukaryotes entail a function (e.g. apatite in bones has a structural role, aragonite or silica in shells provide protection against predators), whereas in prokaryotes this function may be apparent or unknown.

Biomineralization can be associated with microbial respiratory processes. In this process, microorganisms couple the oxidation of organic or inorganic electron donors (e-donor) with the reduction of organic or inorganic electron acceptors (e-acceptor), resulting in a flow of electrons that generates chemical energy (ATP). In certain cases, microbial respiration using metals and metalloids as terminal electron acceptors leads directly or indirectly to solid products such as metal sulfides (AsS, FeS), elemental selenium (Se^0^), elemental sulfur (S^0^), and iron (oxyhydr)oxides. Since it involves various chemical elements, biomineralization serves as a chemical hub in both the oxidative and reductive pathways of several biogeochemical cycles (Staicu and Barton 2024). Therefore, understanding these processes is relevant from fundamental (identifying potential biological functions impacted by certain biominerals in prokaryotes) and applied perspectives (e.g. attractive for industrial applications such as biohydrometallurgy and bioelectrochemical systems).

In this manuscript, we focus on biominerals associated with microbial respiratory processes, including iron oxides, FeS_x_, S^0^, AsS and Se^0^. The main focus of this review is neutrophilic, pure (axenic) microbial cultures involved in the production of the above-listed biominerals. For each biomineral, the article explores thermodynamics, enzymology, and mineralogy, as well as some aspects related to their characterization, environmental distribution and application. These sections are preceded by a brief introduction to anaerobic respiration in prokaryotes aimed at facilitating the reader with a better understanding of the biomineralization process. This article aims to link anaerobic respiration and microbial biomineralization in a framework that integrates the latest updates with the perspectives resulting from this multidisciplinary topic.

## A brief overview of microbial respiration

Microbial respiration is a process in which microbes transform organic and inorganic matter while generating reducing power to produce ATP, the essential energy molecule for all living organisms. In this process, electron acceptors play a crucial role in facilitating energy generation. The reduced cofactors, such as NADH and FADH_2_ generated during catabolic processes, are oxidized through electron transfer pathways to suitable electron acceptors. These can be membrane enzymes or protein complexes present at the inner membrane. This electron flow is linked to the translocation of charge (e.g. electrons or ions), forming an electrochemical gradient across the inner membrane. This gradient creates a proton motive force that drives ATP synthesis by the membrane-bound ATP synthase (Schäfer 2013). This process is in contrast with fermentation, which proceeds in the absence of an external electron acceptor, with cells maintaining redox reactions by transferring electrons to organic intermediates, thereby allowing the production of ATP.

**Figure 1.**
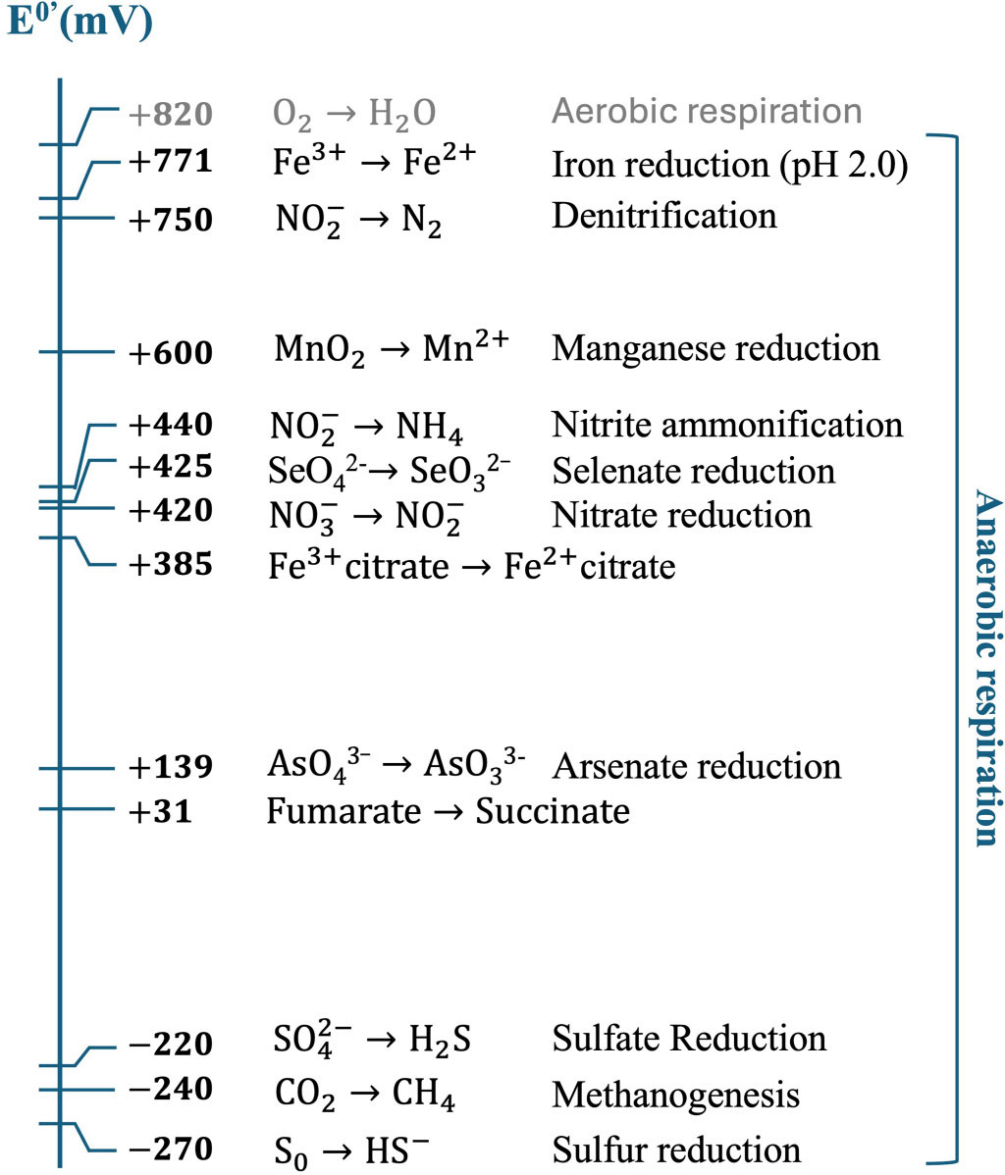
Reduction potential (E^0’^ vs SHE) of electron acceptors used in microbial respiration, emphasizing those used under standard anoxic conditions (Aghababaie et al. 2015). Note that these are reduction potentials under standard conditions (1 M concentration for all reactants and substrates at pH = 0, 25°C and 1 bar). The reaction feasibility under biologically relevant conditions can be determined by the methods outlined in Amend and LaRowe (2019).

While oxygen serves as the primary electron acceptor in aerobic respiration, in the absence of oxygen, microbial respiration relies on alternative electron acceptors that can be utilized intracellularly or extracellularly. These include nitrate (NO_3_^−^), sulfate (SO_4_^2−^), arsenate (AsO_4_^3−^), selenate (SeO_4_^2−^), selenite (SeO_3_^2−^) and metal ions (e.g. Fe(III), Mn(IV)), among others. These inorganic compounds, besides being used in dissimilatory metabolisms to generate energy, can also participate in assimilatory processes, where they are reduced and incorporated into biomass. The ATP yield in anaerobic respiration is less efficient than in aerobic respiration since the terminal electron acceptors used in anaerobic respiration have a lower reduction potential than oxygen (Fig. 1). ATP synthase efficiency is directly related to the electron donor and the terminal electron acceptor, in which the greater difference in reduction potential between the two results in higher energy generation.

Dissolved electron acceptors can enter microbial cells and undergo reduction intracellularly, while solid electron acceptors, such as metal oxides, are reduced extracellularly. For soluble electron acceptors, the initial step of anaerobic respiration is the transport of dissolved electron acceptors across the cell wall, as their terminal reductases are located within the cell. However, the specific transport mechanisms vary depending on the type of electron acceptor and the organism. Although small molecules, such as O_2_ or CO_2_, can easily diffuse across the cell membrane, other compounds require specific cellular machinery to enter the cell. ATP-binding cassette (ABC)-type transporters are the most well-known and well-characterized transporters. These protein complexes couple the energy released from ATP hydrolysis to the translocation of a wide variety of substances in and out of cells (Locher 2009). Once inside the cell, the electrons reduce the electron acceptors, which may involve a single step, or several events catalyzed by different enzymes.

When the electron acceptor is insoluble and cannot permeate the membrane, specific electron transfer pathways are required to transport the electrons outside the cell. This is the case with metal oxides, which are often present as solid minerals (Gralnick and Newman 2007). To overcome this challenge, microorganisms have evolved specialized mechanisms, such as extracellular electron transfer, that facilitate electron exchange between intracellular redox reactions and extracellular electron acceptors (Shi et al. 2016, Kappler et al. 2021). This process can occur through a direct mechanism, where electrons derived from catabolic reactions are transported to cell-surface exposed proteins that directly contact and transfer the electrons to the solid electron acceptor, or through an indirect process using soluble electron shuttles (Costa et al. 2018, Liu et al. 2018). Several electron shuttles have been identified in nature, and microorganisms either produce their own electron shuttles or utilize those available in the environment (Glasser et al. 2017). These electron shuttles (e.g. flavins, quinones, humic substances) facilitate the reduction of solid electron acceptors that cannot easily enter the cell interior (Gralnick and Newman 2007) and, for that reason, they mediate electron transfer from exposed redox proteins to insoluble (and poorly soluble) electron acceptors (Paquete et al. 2014).

## Iron biominerals

### Fe(III) (oxyhydr)oxides

#### Fe minerals in nature and their importance

Fe(III) (oxyhydr)oxides minerals can be both a product and a substrate of microbial respiration (Kappler et al. 2021). Ferrihydrite (FeOOH), lepidocrocite (gamma-FeOOH), goethite (alpha-FeOOH), hematite (alpha-Fe_2_O_3_), and magnetite (Fe_3_O_4_) are abundant Fe mineral phases in the environment. They play a key role in many environmental processes due to their high reactivity, small particle size and the resulting high surface area. This includes the sorption of nutrients (e.g. phosphate and trace metals such as Ni) and toxic metals (e.g. As), she orption of organic carbon which protects it from biodegradation, and the redox transformation of organic and inorganic pollutants (Borch et al. 2010).

#### Abiotic and biotic Fe(II) oxidation

Fe(III)-bearing minerals can be formed at neutral pH abiotically via oxidation of Fe(II) by oxidants such as dioxygen (O_2_) or nitrite (NO_2_^−^), photochemically, i.e. catalyzed by light of different wavelengths, and also enzymatically by phototrophic, nitrate-reducing and microaerophilic microorganisms (Kappler et al. 2021). While the first metabolic group uses photosynthesis to couple Fe(II) oxidation to CO_2_ reduction and biomass formation (i.e. photoferrotrophy), the latter two use respiration with either nitrate or oxygen as electron acceptors for their metabolism, i.e. for energy generation.

The oxidation of Fe(II) by microorganisms at neutral pH by respiration with either nitrate or molecular oxygen as electron acceptor poses several challenges for the microorganisms compared to respiratory oxidation of organic compounds or gases such as hydrogen (H_2_) or methane (CH_4_). While the oxidation of electron donors that are typically dissolved in water can be performed after the uptake of the substrates into the cells, followed by a release of soluble or gaseous compounds (including CO_2_), the respiratory Fe(II)-oxidizers face various physiological challenges. On the one hand, many Fe(II)-bearing substrates are present as (in)soluble minerals (e.g. siderite (FeCO_3_), vivianite (Fe_3_(PO_4_)_2_), magnetite (Fe_3_O_4_), as well as Fe(II)-bearing clays) (Kappler et al. 2021). On the other hand, the oxidation of Fe(II) at neutral pH leads to poorly soluble Fe(III). This oxidized form of Fe precipitates readily as one of the typical Fe(III) (oxyhydr)oxide minerals—the identity mostly depending on the environmental conditions—potentially harming the cellular metabolic activity by precipitating within the cells or at the cell surface (Kappler et al. 2005, Miot et al. 2009a). Different Fe(II)-oxidizers have been shown to deal with these issues in different ways: from extracellular oxidation of Fe(II) minerals, uptake and oxidation of only dissolved Fe^2+^ or organic-complexed Fe(II) (Miot et al. 2009b, Byrne et al. 2016, Peng et al. 2018, Han et al. 2020, Jakus et al. 2021, Zhou et al. 2022) to changes in cell-surface charge or cell surface pH to prevent cell encrustation (Hegler et al. 2010, Saini and Chan 2013). Finally, the group of microaerophilic Fe(II)-oxidizers produces extracellular organic structures (twisted stalks, sheaths) to deposit the Fe(III) minerals away from the cell itself and thus to maintain the cell metabolic activities (Krepski et al. 2013, Laufer et al. 2017, Vigliaturo et al. 2020).

Finally, it should also be mentioned, that the formation of zero-valent iron (Fe(0) has also been described in iron(III)-reducing cultures of the methanic microorganism *Methanosarcina barkeri* although it remains unclear how widespread this activity is and whether it plays a role under more environmentally relevant conditions (Shang et al. 2020).

### Properties of Fe biominerals

**Figure 2.**
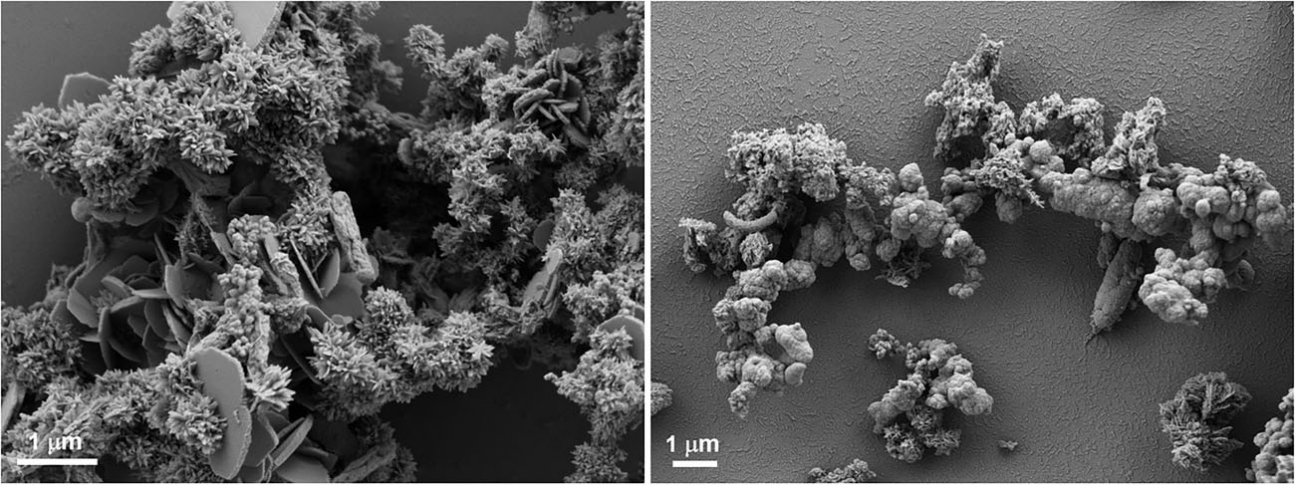
Electron micrographs showing biominerals formed by the phototrophic Fe(II)-oxidizer *Rhodobacter ferroxidans* SW2 (mainly flower-like goethite and flat lepidocrocite structures, left) and the autotrophic nitrate-reducing enrichment culture KS (flower-cluster-like Fe(III) phosphates and Fe(III) oxyhydroxides including ferrihydrite, right). The minerals were identified by X-ray diffraction and Mössbauer spectroscopy. Images were taken by Dr. B. Wan and Dr. J. Shuster.

Biotic Fe(III) (oxyhydr)oxides, formed by microbial Fe(II) oxidation, show distinct properties that distinguish them from their abiotic counterparts (Posth et al. 2014) (Fig. 2). During Fe(II) oxidation, biomolecules, including proteins and carbohydrates, and likely also nucleic acids become associated with the minerals. The positive surface charges of Fe(III) (oxyhydr)oxide minerals at neutral pH facilitate the interactions with the biomolecules that contain negatively charged functional groups or functional groups with free electron pairs, including carboxyl, phosphoryl, and hydroxyl groups. The association of the minerals with these biomolecules influences the properties of the formed minerals. On the one hand, already during the formation of the Fe(III) (oxyhydr)oxides, the organic compounds influence the crystallization of the minerals. Generally, crystal growth is impeded by the competitive sorption of the organic compounds, leading to less crystalline and smaller-sized mineral particles (Posth et al. 2014, Schulz et al. 2022). On the other hand, the surface charge of the minerals can shift from positive to negative affecting interactions with other ions. While the repulsion of silicate has been observed for some iron biominerals (Schad et al. 2022), silicate is generally known to sorb strongly to abiotic Fe(III) (oxyhydr)oxides and to other ferric iron biominerals (Senn et al. 2015, Zhou et al. 2024). Trace metals (such as Ni, and many other rare earth elements) also show different sorption behavior to abiotic and biogenic iron minerals (Eickhoff et al. 2014, Heim et al. 2015, Schmid et al. 2016, Kovalick et al. 2024). Therefore, a deeper investigation into the reactivity of biominerals is required for understanding the environmental behavior of trace metals, nutrients and organic carbon; in particular the reactivity of iron biominerals needs to be studied in more detail. It would be of particular interest to study how the transformation of the iron biominerals under fluctuating redox conditions, both via redox-induced solid-state transformation and dissolution-reprecipitation processes, changes the reactivity and properties of the minerals with regard to their interactions with trace metals, nutrients and organic carbon. Particularly, the high reactivity of mixed-valent ferrous-ferric mineral phases such as green rust or magnetite could be of relevance under such conditions.

The association of the minerals with biomolecules, or even with whole, intact cells (Fig. 2), also affects the density of Fe minerals. While abiotic minerals such as ferrihydrite or goethite have densities of 3.6–4.0 g/cm^3^, a study using different Fe(II)-oxidizing bacteria showed that biotic Fe(III) (oxyhydr)oxide minerals have much lower densities, in the range of 1.6–2.4 g/cm^3^ (Posth et al. 2010). This, in turn, may influence sedimentation behavior of the mineral particles and cell-mineral aggregates, meaning that Fe(III) (oxyhydr)oxide minerals that are formed in the water column of a lake or ocean would have a much longer residence time in the water column and be available for prolonged Fe redox cycling at the chemocline (Schad et al. 2022, Dreher et al. 2025) than their abiotic counterparts (e.g. detrital mineral phases), with potential consequences for their interactions with nutrients, pollutants, and other organisms.

### Application and relevance of iron biominerals

Minerals produced by microorganisms, including iron biominerals, can be used for many interesting future practical applications (summarized recently in Cosmidis 2023). This includes the use of magnetic nanoparticles produced by magnetotactic bacteria for biomedical applications such as magnetically targeted drug delivery, pathogen detection, magnetic hypothermia for the treatment of tumors, or as contrast agents in magnetic imaging techniques as well as for remediation of toxic metals such as Cd or As from groundwater (see references in Caraballo et al. 2022, Cosmidis 2023 and Li et al. 2025). Additionally, very recently a potential role of redox-active iron biominerals in electron storage, i.e. the use as biogeobatteries, has been suggested (Peiffer et al. 2021). In addition, iron-metabolizing microbes play a vital role in recovering valuable metals (such as rare earth elements or even gold or platinum), extracting toxic metals (such as arsenic): (Hohmann et al. 2010, Omoregie et al. 2013, Sowers et al. 2017), and supporting bioremediation (Gadd 2010). Furthermore, iron biominerals have been studied in the context of life on other planets (Price et al. 2022) and the search for evidence for life or certain microbial processes on early Earth (Lin et al. 2017).

### Iron sulfide biominerals

**Figure 3.**
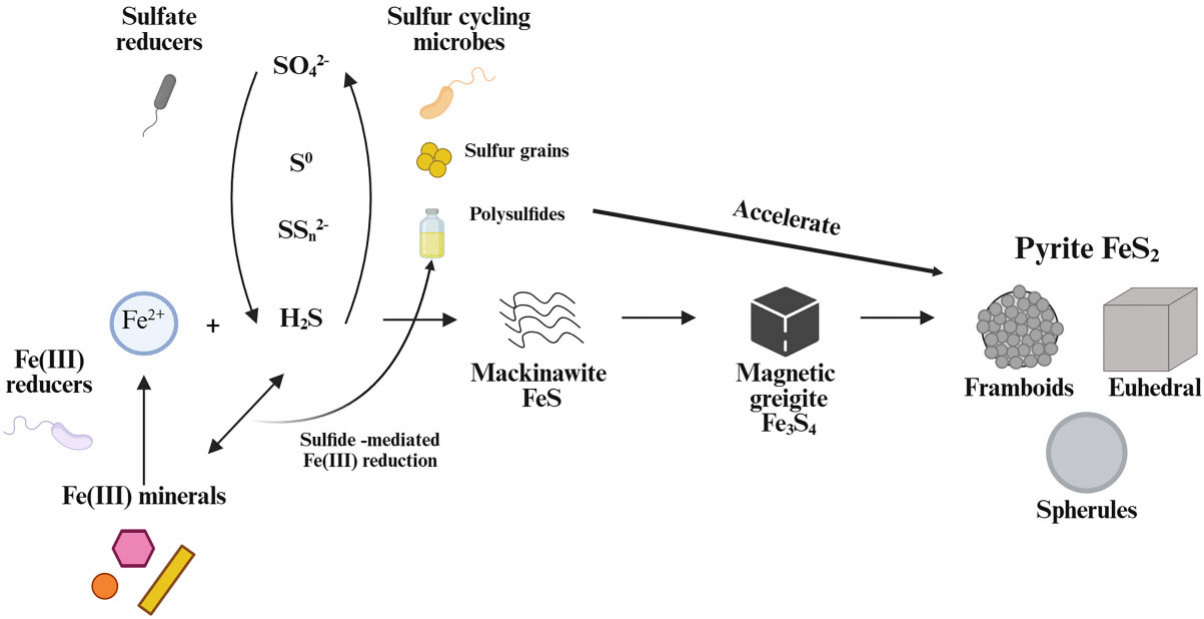
Summary of formation pathways for iron sulfides and the microorganisms involved. Fe(III)-reducers reduce various Fe(III) minerals to dissolved Fe^2+^. The reaction between dissolved Fe^2+^ and H_2_S released from sulfur-cycling microorganisms results in mackinawite precipitation. Its transformation to greigite and pyrite is accelerated in the presence of various intermediate sulfur species (S^0^, SS_n_^2^) produced by sulfur-cycling microorganisms and through the abiotic reaction between H_2_S and Fe(III) minerals.

Microbial respiration of sulfur (S) species and ferric iron (Fe(III)) produces reduced sulfide (H_2_S) and ferrous iron (Fe(II)), which precipitate together to form a variety of iron sulfides such as mackinawite (FeS), greigite (Fe_3_S_4_) and pyrite (FeS_2_) via BIM (Fig. 3). The production of sulfide is driven by various sulfide producing microorganisms (SPM), which catalyze the reduction of sulfate (SO_4_^2−^) or disproportionation of sulfur species such as elemental sulfur (S^0^) and thiosulfate (S_2_O_3_^2−^) (Jørgensen et al. 2019). Among these, sulfate reduction is the most important, with sulfate reducing microorganisms thought to constitute >90% of the sulfide source at low temperatures globally (Rickard et al. 2017). Meanwhile, Fe(III) reducing microorganisms (FeRM) couple the reduction of Fe(III) with the oxidation of organic carbon, dihydrogen (H_2_) or methane (CH_4_) (Kappler et al. 2021). SPM and FeRM co-exist in a variety of anoxic habitats, such as in sediments and stratified water columns. The formation of biogenic iron sulfides via BIM by these microorganisms is tied to the fate of carbon, oxygen, nitrogen, phosphorus and various trace metals at both short-term and geological time scales (Mansor et al. 2025).

Enzymes involved in the production of sulfide and Fe(II) can be easily detected from (meta)omic datasets using functional annotations via hidden Markov models (Garber et al. 2020). For SPM, these include (1) the set of sulfate adenylyltransferase (Sat), APS reductase (Apr) and dissimilatory sulfate reductases (DsrAB and DsrMKJOP) for sulfate reduction, (2) sulfur reductase (Sre) or NAD(P)H sulfur oxidoreductase (Nsr) for S^0^ reduction, and (3) sulfur oxygenase reductase (Sor), thiosulfate-thiol sulfurtransferase (RDL2), cytochrome *c_3_* or membrane-bound quinone-dependent thiosulfate reductase (Phs) for the disproportionation of S^0^ or thiosulfate (Zhou et al. 2025). For FeRM, enzymes such as (1) cytoplasmic membrane-associated tetrahaem c-type cytochrome (CymA), (2) metal-reducing associated genes (MtrCAB) and (3) outer membrane cytochromes (OmcF, OmcS and OmcZ) are indicators of Fe(III) reduction (Garber et al. 2020).

Mackinawite (FeS) is the first product to form upon the reaction between iron and sulfide (Rickard and Luther 2007). Individual crystallites of mackinawite are typically less than 20 nm in size, but it can aggregate in solution to form visible black clumps (Picard et al. 2018). Several studies have investigated mackinawite formation by sulfate reducers in the context of pollutant remediation, carbon sequestration and biosignatures (Picard et al. 2016, 2019, 2021, Ikogou et al. 2017, Stanley and Southam 2018, Mansor et al. 2019a,b, Nabeh et al. 2022). Biogenic mackinawite is typically larger, displays higher aggregation extent, and is associated with more organic matter compared to abiogenic mackinawite precipitated in the absence of microorganisms (Picard et al. 2018, 2019, 2021, Mansor et al. 2019a, Nabeh et al. 2022). The order of addition—iron first or sulfide first—affects the association between cells and mackinawite. When Fe is added first to the culture medium, positively charged Fe^2+^ binds to the negatively charged cell wall. Sulfide produced by the sulfate reducers then precipitates with Fe^2+^ on the cell wall, leading to encrustation and likely decreased cell viability. When sulfide is already present, Fe^2+^ added later immediately reacts with sulfide, causing precipitation in the extracellular space (Picard et al. 2018, 2021); Nabeh et al. 2022. Both scenarios could be envisioned in the environment, with impacts on microbial ecology and biosignature detection.

Besides sulfate reducers, recent studies have started to investigate the role of other microorganisms in precipitating mackinawite. For example, *Geobacter sulfurreducens* is a well-known Fe(III)-reducer that can also reduce S^0^ to sulfide. In the co-presence of Fe(III) and sulfide, mackinawite is formed with implications for phosphorous bioavailability (Bronner et al. 2023) and for sulfur and iron-based bioremediation technology (Liu et al. 2023). Similarly, *Shewanella* species produce mackinawite when grown in the co-presence of thiosulfate and Fe(III), with a noticeable enhancement on mineral-mediated extracellular electron transfer that is being considered for application in microbial fuel cells (Nakamura et al. 2010, Jiang et al. 2014, Kondo et al. 2015). The hyperthermophilic archaeon *Thermococcus kodakarensis* produces mackinawite when grown on S^0^, which is of importance for iron sulfide formation and life detection near hydrothermal vents (Gorlas et al. 2018, 2022, Truong et al. 2023, 2024).

Greigite (Fe_3_S_4_) is a mixed-valent Fe(II)-Fe(III) sulfide mineral that can form upon mackinawite aging. Intracellular greigite is known to be formed by magnetotactic bacteria together with magnetite (Fe_3_O_4_) via BCM, where these magnetic nanominerals are used by the microorganisms to aid in navigating the Earth’s magnetic field (Amor et al. 2020). Reports of extracellular greigite formation via BIM in microbial cultures are relatively rare (Picard et al. 2018, Mansor et al. 2019a, Gorlas et al. 2022, Bronner et al. 2023, Sekerci et al. 2025). The transformation of mackinawite to greigite requires an oxidant, which could be low amounts of oxygen, protons or polysulfides (Rickard and Luther 2007, Mansor et al. 2025). This transformation is faster in the presence of sulfate reducers, with the mineral being detectable within 6 months, in comparison to abiotic controls that showed no transformation of mackinawite to greigite (Picard et al. 2018, Mansor et al. 2019a). In cultures of *G. sulfurreducens*, greigite is detected as early as 21 days (Bronner et al. 2023). Extracellular greigite formation is even faster in the presence of the hyperthermophilic *T. kodakarensis*, becoming detectable within a few days. These greigite nanoparticles occur in close proximity to cells and S^0^-containing vesicles (Gorlas et al. 2018, 2022, Truong et al. 2023). Overall, differences between extracellular biogenic and abiogenic greigite are relatively understudied.

Over time, and under the right environmental conditions, mackinawite and greigite can transform to pyrite (FeS_2_). Pyrite is the most abundant iron (di)sulfide mineral on Earth, where it is present in the form of striking framboids in addition to euhedral and spherical morphology (Mansor et al. 2025). The burial of pyrite is one of the most important factors affecting the oxygen balance in the ocean-atmosphere system (Berner 1989, Canfield 2005, Canfield and Farquhar 2009). Strictly, pyrite is an iron disulfide mineral, with sulfur having –1 redox state instead of –2. Hence, pyrite formation is driven by the availability of polysulfides (SS*_n_*^2−^; where *n* is the number of sulfur atoms in a chain). Polysulfides are formed when H_2_S is oxidized by the following chemical species, listed in the order of the reaction kinetics: MnO_2_ > O_2_ > Fe(III) > S^0^ (Avetisyan et al. 2021).

Pyrite formation in cultures of sulfate reducers is rare (Picard et al. 2016), which can be explained mostly by cultivation conditions employing only Fe^2+^ and sulfide, thus excluding any oxidants that can generate polysulfides. Another interesting possibility is that any formed pyrite is immediately reductively dissolved by sulfate reducers, thus evading detection (Boyd and Payne 2025). In cultures containing Fe(III) minerals, S^0^ or mixtures of microbial species capable of cycling Fe and S between redox states (compiled in Mansor et al. 2025), pyrite can be formed at a rate of 0.01-5 mmol/L/day, which is comparable to the rates observed in nature (Mansor et al. 2025). Intriguingly, biogenic pyrite formed in cultures adopts either a spherical (Berg et al. 2020, Duverger et al. 2020, Truong et al. 2023) or euhedral morphology (Thiel et al. 2019, Allen et al. 2021). Natural spherical pyrite has rarely been reported from a microbial mat in the hypersaline Dead Sea (Thomas et al. 2016) and more recently near a hydrothermal vent. Given the lack of pyrite framboids in laboratory cultures, observations of natural framboids cannot be interpreted as a biosignature, especially since many abiotic mechanisms are known (Ohfuji and Rickard 2005).

## Elemental sulfur (S^0^)

### Sulfur occurrence in the environment

**Figure 4.**
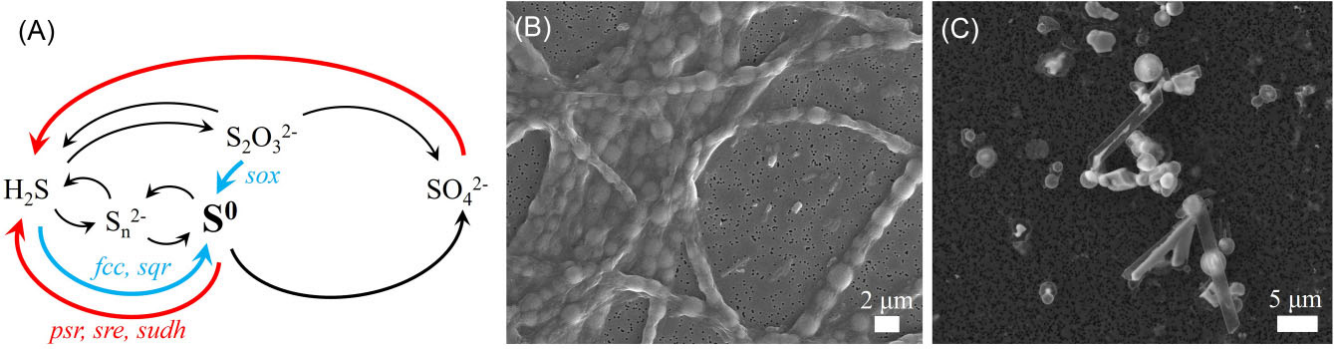
(A) Simplified biogeochemical sulfur cycle, illustrating microbial respiration processes (in red) as well other microbially mediated processes conducing to the formation (in blue) and consumption of S^0^. Abbreviations of genes mentioned in the text are shown in italics. Note that sulfur intermediates such as sulfite or tetrathionate have been omitted. (B) Intracellular S^0^ globules within *Thiothrix sp*. filamentous cells (SEM image by Chrissie Nims). (C) Extracellular S^0^ globules and rods produced by *Sulfuricurvum kujiense* (SEM image by Wang Shuo).

Elemental or zero-valent sulfur (S^0^) is formed in the environment through the re-oxidation of reduced sulfur species (e.g. sulfide) produced by dissimilatory sulfate reduction (Fig. 4A), being considered an indirect product of anaerobic microbial respiration. Once formed, microbial S^0^ does not always accumulate in the environment. Whether it is a transient or the final product of microbial sulfide oxidation varies depending on the composition of the microbial community and local environmental conditions. In marine sediments, S^0^ is mainly found in the first few centimeters below the sediment surface, and at relatively low concentrations (<6.5 µmol/cm^3^ in euxinic sediments, but typically closer to 1 µmol/cm^3^) (Jørgensen 2021). Elemental sulfur is most abundant in marine environments with intense sulfate reduction rates (Zopfi et al. 2004), where the microbial oxidation of reduced sulfur compounds to S^0^ is likely to outpace S^0^ consumption by different metabolic processes. Other environments where biogenic S^0^ may accumulate include salt marshes, hydrothermal sites, sulfidic springs, or caves (Taylor et al. 1999, Kamyshny and Ferdelman 2010, Hamilton et al. 2015, Koeksoy et al. 2018). In some rare cases, S° can form conspicuous and widespread deposits, for instance at the surface of an Arctic glacier fed by a supraglacial sulfidic spring system (Borup Fiord Pass, Ellesmere Island, Canada; Gleeson et al. 2010, Trivedi et al. 2020), or S^0^ plumes forming in surface waters above oceanic oxygen minimum zones (Ohde et al. 2007, Lavik et al. 2009).

### Sulfur biomineralization

Sulfide can be oxidized to S^0^ abiotically through chemical oxidation by oxygen or oxidized metals (Fe(III) and Mn(III, IV)), or biologically through different dissimilatory metabolic pathways. These include prokaryotes performing anoxygenic photosynthesis, using reduced sulfur compounds as electron donors for CO_2_ fixation in the presence of light (e.g. green and purple sulfur bacteria). On the other hand, chemotrophic S-oxidizing bacteria derive energy for CO_2_ fixation from the oxidation of reduced sulfur using either oxygen, nitrate or Mn(IV) oxides (they are classically called “colourless” sulfur bacteria due to their lack of photosynthetic pigments) (Dahl 2020a,b). Under low-temperature conditions, biological sulfide oxidation rates are typically more than three orders of magnitude faster than abiotic ones (Luther et al. 2011), suggesting that most low-temperature (non hydrothermal) S^0^ in nature has a biological origin. The main enzymes involved in sulfide oxidation are the FAD-containing flavocytochrome c (FccAB) and sulfide: quinone oxidoreductases (SQR). S^0^ can also be formed through microbial oxidation of thiosulfate, catalyzed by enzymes of the SOX system (Dahl 2020b).

Biogenic sulfur is formed as a biomineral either intracellularly or extracellularly (Kleinjan et al. 2003). Intracellularly, S^0^ takes the form of spherical globules (Dahl 2020b) (Fig. 4B), where sulfur may be present as nano-crystalline or amorphous cyclooctasulfur (S_8_), occasionally associated with linear forms of polymeric sulfur such as polysulfides or polythionates (Pasteris et al. 2001, Prange et al. 2002, Prange 2008, Nims et al. 2019). Depending on microbial species, intracellular S^0^ may be located within invaginations of the cytoplasmic membrane, or form inclusions in the cytoplasm with no connection to the cytoplasmic membrane (Dahl 2020b). In some cases (e.g. *Beggiatoa, Thiothrix*, or *Thiovulum*), proteins forming an envelope around the S^0^ biominerals have been observed. The best studied of these structural proteins are the strongly hydrophobic proteins SgpA, SgpB, SgpC present in the purple sulfur bacterium *Allochromatium vinosum*, which have been shown to play essential roles in intracellular S^0^ accumulation (Brune 1995, Prange et al. 2004). A fourth sulfur globule protein, SgpD, has been discovered more recently and plays a yet unclear role in the formation of S^0^ inclusions (Kümpel et al. 2023).

Extracellular S^0^ biominerals can form at the cell surface or away from the cells and adopt spherical or more irregular morphologies (Fig. 4C). It may be composed of different cyclooctasulfur polymorphs, namely the thermodynamically stable orthorhombic α-S_8_ (sometimes in a nanocrystalline form), or the metastable monoclinic β- and γ-S_8_ (Cron et al. 2019, Marnocha et al. 2019). Metastable forms of S^0^ are allowed to form and persist in the environment through stabilizing interactions with microbially derived organics. Organic envelopes have indeed been observed at the surface of extracellular S^0^ biominerals formed in laboratory cultures (Kleinjan et al. 2005, Cron et al. 2019, Marnocha et al. 2019) as well as in natural biofilms (Cron et al. 2021). These organic coatings are usually composed of polysaccharides and proteins, while in at least some species they are lipidic, being derived from outer membrane vesicles (Li et al. 2020).

### Sulfur utilisation

Most organisms that form S^0^ biominerals have the metabolic capacity to complete the oxidation to sulfate for energy generation. In those cases, S^0^ usually serves an energy storage function (Cosmidis and Benzerara 2022), being produced and stored under favorable environmental conditions and then used as an electron donor when conditions change. This process is observed in S-oxidizers that produce S^0^ both intra- and extracellularly. For instance, *Beggiatoa* oxidizes sulfide to intracellular S^0^ under high-sulfide conditions, while under low-sulfide conditions this stored S^0^ is oxidized to sulfate (Berg et al. 2014). Similarly, the green sulfur bacterium *Chlorobaculum tepidum* forms extracellular S^0^ globules during sulfide oxidation, and then uses this biogenic S^0^ as an electron donor for photosynthesis when sulfide is exhausted (Hanson et al. 2016). The utilization of extracellular S^0^ may require cell attachment to the particles, via the involvement of thiol groups interacting with S^0^ at the cell surface. It can also occur at distance, through pili-assisted extracellular electron transport, or extracellular conversion of S^0^ to soluble polysulfides or thiosulfate. The complex molecular systems involved in intracellular sulfur trafficking and oxidation have been described elsewhere (Jia et al. 2024) and are outside the scope of this review.

Under certain conditions, S^0^ formed by purple sulfur bacteria can serve as an electron acceptor for anaerobic respiration, leading to its reduction back to sulfide (Fig. 4A). For instance, *Allochromatium vinosum* uses intracellular S^0^ as a source of reducing power for anoxygenic photosynthesis in the absence of sulfide, releasing sulfate, while in the dark, S^0^ is used as an electron acceptor, releasing sulfide (Mas and Gemerden 1995). S^0^ reduction to sulfide has also been proposed for non-photosynthetic sulfur bacteria. *Thiovulum*, which stores intracellular S^0^ under aerobic conditions, can anaerobically reduce its stored S^0^ by respiration, possibly with formate as the electron donor or via sulfur disproportionation (Marshall et al. 2012). S^0^ respiration to sulfide was also observed in the anaerobic growth of *Beggiatoa* in the presence of acetate (Nelson and Castenholz 1981).

The ability to respire S^0^ (or more rarely, disulfide) using H_2_ or organic substrates as electron donors is also widespread among non S^0^-biomineralizing bacteria and archaea, most of which are hyperthermophilic (Hedderich et al. 1998). The molecular mechanisms involved in S^0^ respiration remain to be elucidated, but three main enzymes have been identified: polysulfide reductase (Psr), sulfur reductase (Sre) and sulfide dehydrogenase (Sudh) (Ma and Adams 1994, Hedderich et al. 1998, Florentino et al. 2017, Wang et al. 2023). Beyond its role in respiration, sulfur is used in some fermentative archaea in a process known as sulfhydrogenesis, whereby S^0^ or polysulfides act as a sink for excess electrons to reoxidize reduced ferredoxin accumulated during fermentation, thereby balancing the cell’s redox state (Ma and Adams 1994).

## Arsenic and selenium

The presence of arsenic and selenium in the environment is on the rise due to various anthropogenic activities such as fossil fuel burning for energy production, metal and crude oil refining, agriculture etc., exerting a significant negative impact on terrestrial and aquatic ecosystems as well as on human communities (Ganie et al. 2024, Vinceti et al. 2024).

### The thermodynamics of As and Se respiration

The use of selenium (Se) and arsenic (As) oxyanions: selenate/Se^6+^ (SeO_4_^2−^), selenite/Se^4+^ (SeO_3_^2−^) and arsenate/As^5+^ (AsO_4_^3−^) as terminal electron acceptors in microbial anaerobic respiration began to be documented by the end of the 1980s and early 1990s (Stolz et al. 2006). Interestingly, both elements were historically known as potent toxicants for bacteria, so their role in anaerobic respiration was surprising. All oxyanions provide significant amounts of cellular energy for bacteria capable of utilizing it (Fig. 1; Eqs. 1-3) (Staicu and Barton 2021).


(1) \begin{eqnarray*}
&& {{{\mathrm{C}}_3}{{\mathrm{H}}_5}{{\mathrm{O}}_3}^ - + 2{\mathrm{Se}}{{\mathrm{O}}_4}^{2 - } \to {{\mathrm{C}}_2}{{\mathrm{H}}_3}{{\mathrm{O}}_2}^ - + 2{\mathrm{Se}}{{\mathrm{O}}_3}^{2 - } + {\mathrm{HC}}{{\mathrm{O}}_3}^ - + {{\mathrm{H}}^ + }}\\
&&\quad {\Delta {G^{0^{\prime}}} = - 343.1\,{\mathrm{kJ}}\,{\mathrm{mo}}{{\mathrm{l}}^{ - 1}}{\mathrm{lactate}}({{\mathrm{C}}_3}{{\mathrm{H}}_5}{{\mathrm{O}}_3}^ - )}
\end{eqnarray*}

(2) \begin{eqnarray*}
&& {{{\mathrm{C}}_3}{{\mathrm{H}}_5}{{\mathrm{O}}_3}^ - + {\mathrm{Se}}{{\mathrm{O}}_3}^{2 - } + {{\mathrm{H}}^ + } \to {{\mathrm{C}}_2}{{\mathrm{H}}_3}{{\mathrm{O}}_2}^ - + {\mathrm{S}}{{\mathrm{e}}^0} + {\mathrm{HC}}{{\mathrm{O}}_3}^ - + 2{{\mathrm{H}}_2}{\mathrm{O}}}\\
&&\quad {\Delta {G^{0^{\prime}}} = - 529.5\,{\mathrm{kJ}}\,{\mathrm{mo}}{{\mathrm{l}}^{ - 1}}{\mathrm{lactate}}( {{{\mathrm{C}}_3}{{\mathrm{H}}_5}{{\mathrm{O}}_3}^ - })}
\end{eqnarray*}

(3) \begin{eqnarray*}
&& {{{\mathrm{C}}_3}{{\mathrm{H}}_5}{{\mathrm{O}}_3}^ - + 2{\mathrm{HAs}}{{\mathrm{O}}_4}^{3 - } + 4{{\mathrm{H}}^ + } \to {{\mathrm{C}}_2}{{\mathrm{H}}_3}{{\mathrm{O}}_2}^ - + 2{\mathrm{HAs}}{{\mathrm{O}}_2} + {\mathrm{C}}{{\mathrm{O}}_2}}\\
&&\quad {+\, 3{{\mathrm{H}}_2}{\mathrm{O}}} {\Delta {G^{0^{\prime}}} = - 172\,{\mathrm{kJ}}\,{\mathrm{mo}}{{\mathrm{l}}^{ - 1}}{\mathrm{lactate}}( {{{\mathrm{C}}_3}{{\mathrm{H}}_5}{{\mathrm{O}}_3}^ - })}
\end{eqnarray*}

It is noteworthy to point out that both SeO_4_^2–^ (reduced to SeO_3_^2−^) and SeO_3_^2−^ (reduced to solid Se^0^) accept electrons in this process, whereas only AsO_4_^3−^ (reduced to arsenite/As^3+^, AsO_3_^3−^) fulfills this function. This means that Se not only provides more cellular energy compared to As, but it is also more chemically versatile in anaerobic respiration (Eqs. 1-3). Energetically speaking, with lactate as electron donor, SeO_4_^2−^ provides twice more energy than AsO_4_^3−^, while the reduction of SeO_3_^2−^ to solid Se^0^ generates over three times more. Gibbs Free Energy (*ΔG*^0'^) is the free energy change determined under standard conditions. While the optimum growth of most arsenic- and selenium-respiring bacteria cultivated in the laboratory occurs at non-standard conditions (e.g. 28–30°C, pH ∼ 7–7.5), the standard free energy change values are regularly employed in scientific literature of bacterial energetics.

### Biogenic Se^0^

Se^0^ biomineralization in bacteria results from detoxification and respiratory processes, and it generally produces amorphous minerals (Ruiz-Fresneda et al. 2023). In the case of detoxification, Se^0^ particles form intracellularly, following the uncontrolled cellular uptake of toxic Se oxyanions (Ni et al. 2015). On the other hand, the respiratory-related biomineralization of Se is more complex and raises a number of unresolved questions. A major question is the fate of SeO_3_^2−^ produced via the respiration of SeO_4_^2−^ in the periplasmic space. Since SeO_3_^2−^ is toxic, the best option for bacteria would be to export it away from the cell.

**Figure 5.**
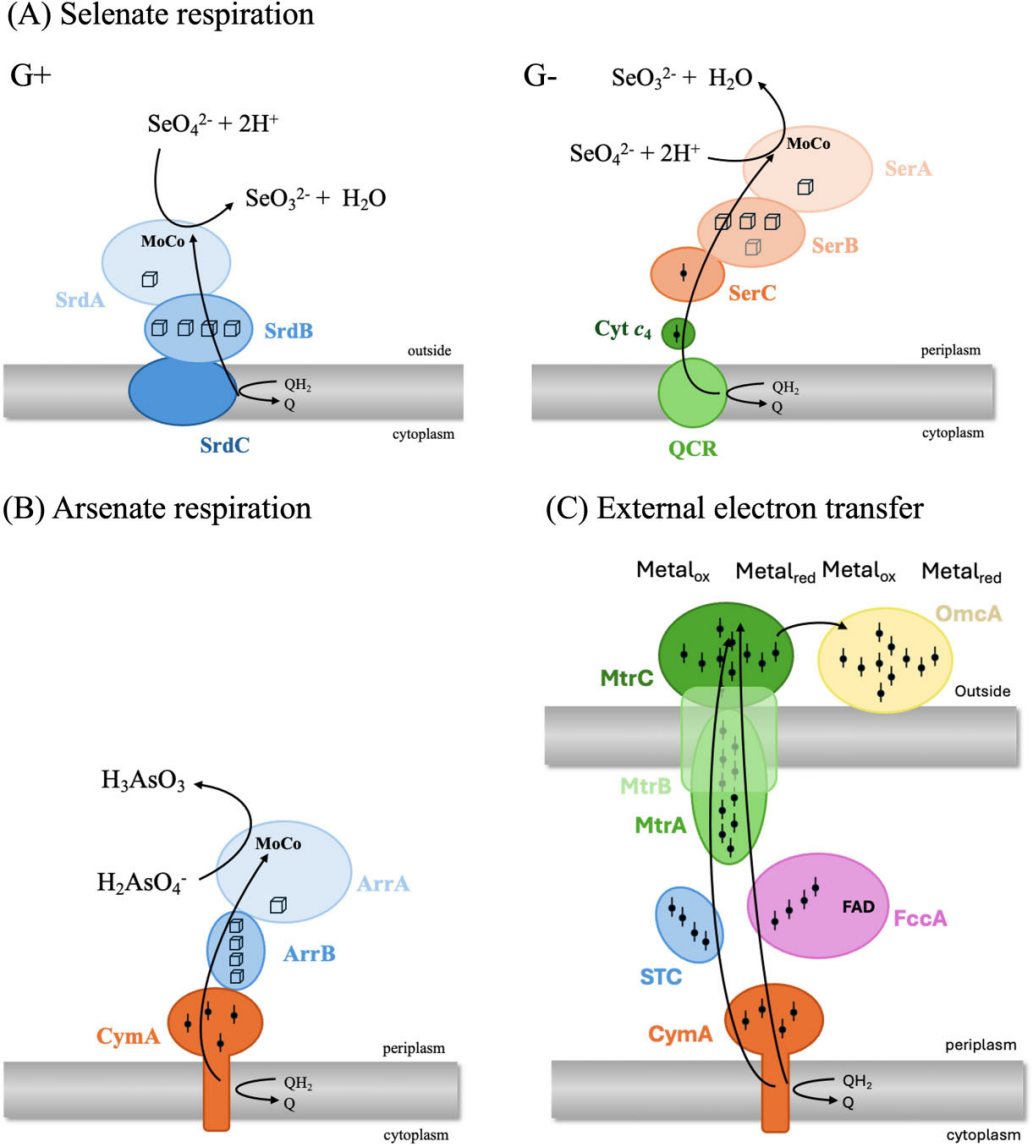
Reductive pathways of various electron acceptors, with the proteins involved: (A) Selenate respiration (Kuroda et al. 2011); (B) Arsenate respiration (Glasser et al. 2018); (C) External electron transfer (Lemaire et al. 2020). **Acronyms**: ArrAB: Arsenate reductase; CymA: Tetraheme c-type cytochrome; Cyt c4: Cytochrome c4; FAD: Flavin Adenine Dinucleotide (electron carrier); FccA: Fumarate reductase; G+: Gram positive; G-: Gram negative; MoCo: Molybdenum cofactor; MtrA: Periplasmic decaheme c-type cytochrome; MtrB: Multiheme cytochrome; MtrC: Outer membrane cytochrome; OmcA: Outer membrane cytochrome; Q: Ubiquinone; QH_2_: Ubiquinol; QCR: Quinol-cytochrome c reductase; STC: Small tetraheme cytochrome; SerABC: Selenate respiratory reductase from *Thauera selenatis*; SrdABC: Selenate respiratory reductase from *Bacillus selenatarsenatis* SF-1.

*Thauera selenatis* (β-proteobacteria; Gram-negative/G-) was the first bacterium described to perform anaerobic respiration using selenate as an e-acceptor (Fig. 5B). It was isolated by Joan Macy and coworkers in 1989 from a bioreactor setting where Se-laden effluents were treated biologically. The reduction of selenate to selenite is coupled with the oxidation of acetate (e-donor) to CO_2_ and intracellular polyhydroxybutyrate (PHB) granules (Macy et al. 1993). In *Thauera selenatis*, the respiration of selenate is driven by the periplasmic selenate respiratory enzyme SerABC (Schröder et al. 1997). SerABC is a trimeric molybdoenzyme with three homologous subunits: Ser A, which is the catalytic subunit and coordinates a molybdopterin cofactor; Ser B, an iron-sulfur protein rich in cysteine residues, with [3Fe–4S] and [4Fe–4S] clusters, and Ser C which contains a *b*-type cytochrome with a standard reduction potential of +234 mV (Schröder et al. 1997).

The complex SrdBCA was identified as the main player in selenate reduction in Gram-positive bacteria. Because Gram-positive/G+ bacteria lack an outer membrane and a periplasmic space, it is assumed that SrdBCA is membrane-bound (Fig. 5B) (Staicu and Barton 2021). This enzyme, isolated from *Bacillus selenatarsenatis* SF-1, is anchored in the membrane by its SrdC subunit, while SrdA (the catalytic subunit containing a MoCo cofactor) and SrdB are located outside the cell. Due to this structure, the Se^0^ biomineralization process is likely to occur in the extracellular environment/vicinity of the cell wall. Similar to *Thauera selenatis*, an in-depth electron microscopy study is also much needed for Gram-positive bacteria capable of respiring selenate.

Selenite respiratory reductase has so far only been identified in *Bacillus selenitreducens* MLS10. This is encoded by an operon containing six genes: *srrA* (catalytic subunit of 80 kDa, with a TAT leader sequence and one putative 4Fe-4S binding site), *srrB* (small subunit of 17 kDa with 4 putative 4Fe-4S binding sites), *srrC* (43 kDa, anchoring subunit), *srrD* (24 kDa, chaperone protein), *srrE* (38 kDa) and *srrF* (45 kDa, rhodanese-domain containing proteins) (Wells et al. 2019).

**Figure 6.**
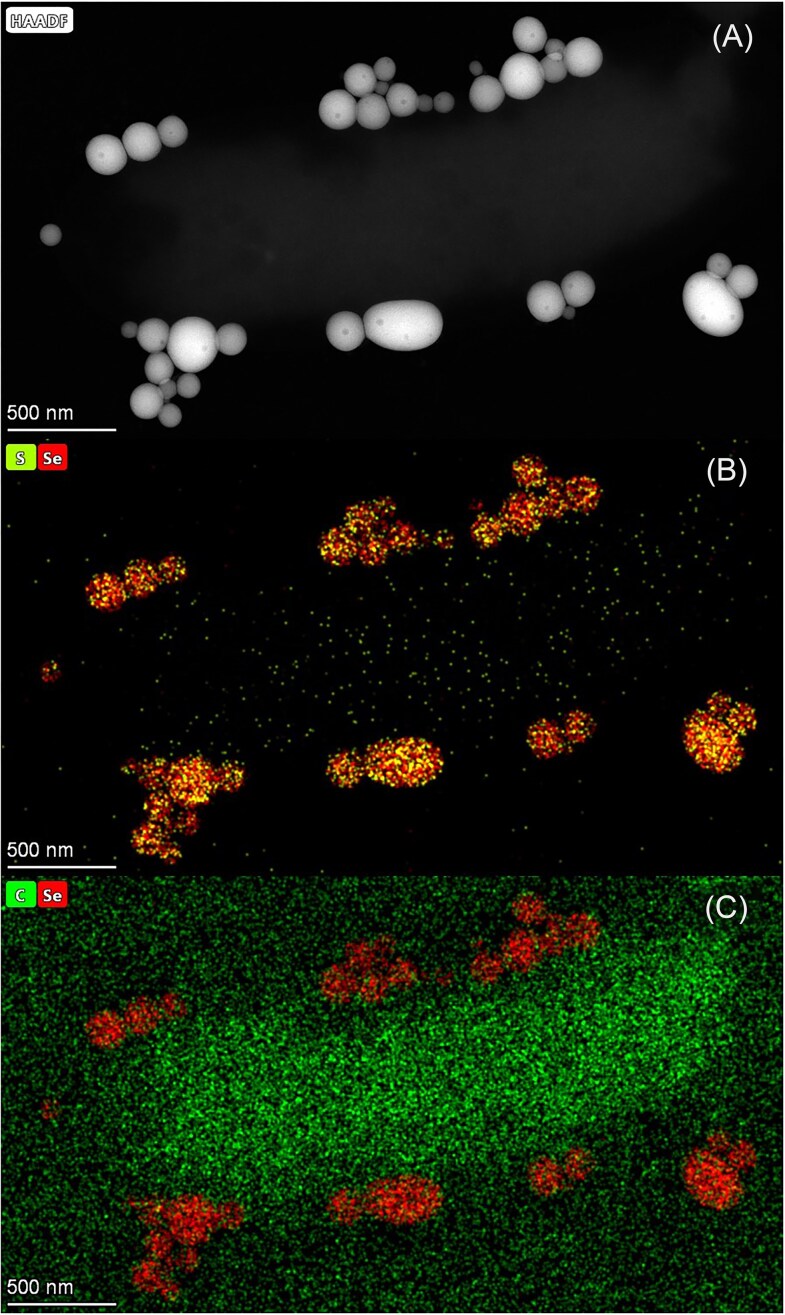
Biogenic Se^0^ produced by *Shewanella* sp. O23S. Micrographs with the corresponding EDS elemental maps obtained from bacterial cells and biomineralization products: A) Scanning transmission electron microscopy (STEM mode); B) Se elemental mapping; C) Sulfur elemental mapping (Staicu et al. 2022a).

A special case is extracellular respiration, where the electrons are routed in the periplasm to the cell wall anchoring the reductase (e.g. Mtr pathway from *Shewanella oneidensis* MR-1). The Mtr pathway contains five primary protein components: MtrC, STC, MtrA, MtrB, and CymA (Shi et al. 2007), MtrC and OmcA being located outside/in the vicinity of the cell wall (Fig. 5C). It has been demonstrated that the fumarate reductase FccA can replace STC in this pathway, being also involved in extracellular electron transfer (Fonseca et al. 2013). While the mechanisms of extracellular respiration is well documented for dissimilatory iron reduction in the model organisms *Shewanella oneidensis* and *Geobacter sulfurreducens*, as well as in other organisms (Shi et al. 2016, Paquete et al. 2022), their involvement in the reduction of other solid compounds is less understood. Extracellular respiration has been well documented in the *Shewanella* and *Geobacter* genera (Gralnick and Newman 2007). An interesting strain of Shewanella that can respire As and Se oxyanions is *Shewanella* sp. O23S (related to *Shewanella baltica*). This strain was isolated from a former gold mine in Poland (Drewniak et al. 2015) and its genome was sequenced and analyzed (Uhrynowski et al. 2019). Notably, *Shewanella* sp. O23S can reduce selenite and selenate to Se^0^ under both oxic and anoxic conditions. Anaerobically, the biomineralization process produces polydisperse, globular Se^0^ particles attached to the surface of the bacterial cells, indicative of extracellular respiration (Fig. 6) (Staicu et al. 2022a). For an in-depth presentation of the molecular aspects of selenium respiration, the reader is referred to Wells and Stolz (2020) and Staicu and Barton (2021).

Transmission electron microscopy (TEM) coupled with Energy-dispersive X-ray spectroscopy (EDS) identified Se and S co-occurring in various ratios. This may indicate a potential contribution of S to the stability of biogenic Se^0^. X-ray Diffraction (XRD) and Raman analysis identified amorphous Se^0^ biominerals (Staicu et al. 2022a). When the surface charge (zeta potential) of the control incubation (nitrate was used as e-acceptor) is compared with that of solutions containing biogenic Se^0^, a marked difference is observed. The zeta potential value of the control is around 0 mV, while the values of Se^0^ biomineral solutions drop to –20 to –30 mV. This may indicate an active accumulation of proteins during the biomineralization process (Staicu et al. 2017). *Shewanella* sp. O23S has also been tested in a bioremediation approach using real metal-rich industrial effluents (Staicu et al. 2015), showing promising results for the removal of As, Se and various metals (Staicu et al. 2022b).

### Biogenic AsS

Despite its toxicity, arsenic can be used by certain bacteria to sustain growth. Microbial respiration using AsO_4_^3−^ as an e-acceptor was discovered around 1994, when strain MIT-13 (Ahmann et al. 1994), later classified as *Sulfurospirillum arsenophilum* (Stolz et al. 1999), was isolated from Aberjona watershed (Boston, Massachusetts), a heavily urbanized river historically polluted with metals and arsenic. Later, it was demonstrated that arsenate reduction is accomplished by an operon whose structure varies across bacteria, featuring different anchoring sub-units (e.g. ArrC, CymA), chaperone proteins (ArrD) and regulatory elements (Fig. 5D). The core enzyme (Arr) is highly conserved, containing the large catalytic subunit (ArrA) and the smaller iron-sulfur cluster subunit (ArrB) (Krafft and Macy 1998). The arsenate reductase from *Shewanella* sp. ANA-3 is a heterodimer (131 kDa): ArrA (95 kDa) and ArrB (27 kDa). It contains one molybdenum (Mo) atom, four sulfur (S) atoms associated with a bis-molybdopterin guanine dinucleotide cofactor, and several [4Fe-4S] clusters (Glasser et al. 2018). It shows no activity in the presence of alternative electron acceptors such as antimonite, nitrate, selenate, and sulfate (Malasarn et al. 2008).

*Sulfurospirillum arsenophilum* uses lactate as e-donor and the respiratory enzyme is located in the periplasmic space. A model of arsenate respiration in Gram-negative bacteria is presented in Fig. 6D. The respiration of arsenate does not lead to the formation of arsenic biominerals directly, unlike in the case of selenate and selenite respiration. Both arsenate and arsenite are water soluble, but the generation of As(III) via anaerobic respiration can be coupled with highly reactive hydrogen sulfides (H_2_S), yielding AsS biominerals. H_2_S is a byproduct of various metabolic processes, such as sulfate respiration or cysteine desulfurization, and is widely present in nature. For a more comprehensive presentation of this process, the reader is referred to Stolz et al. (2006) and Glasser et al. (2018).

**Figure 7.**
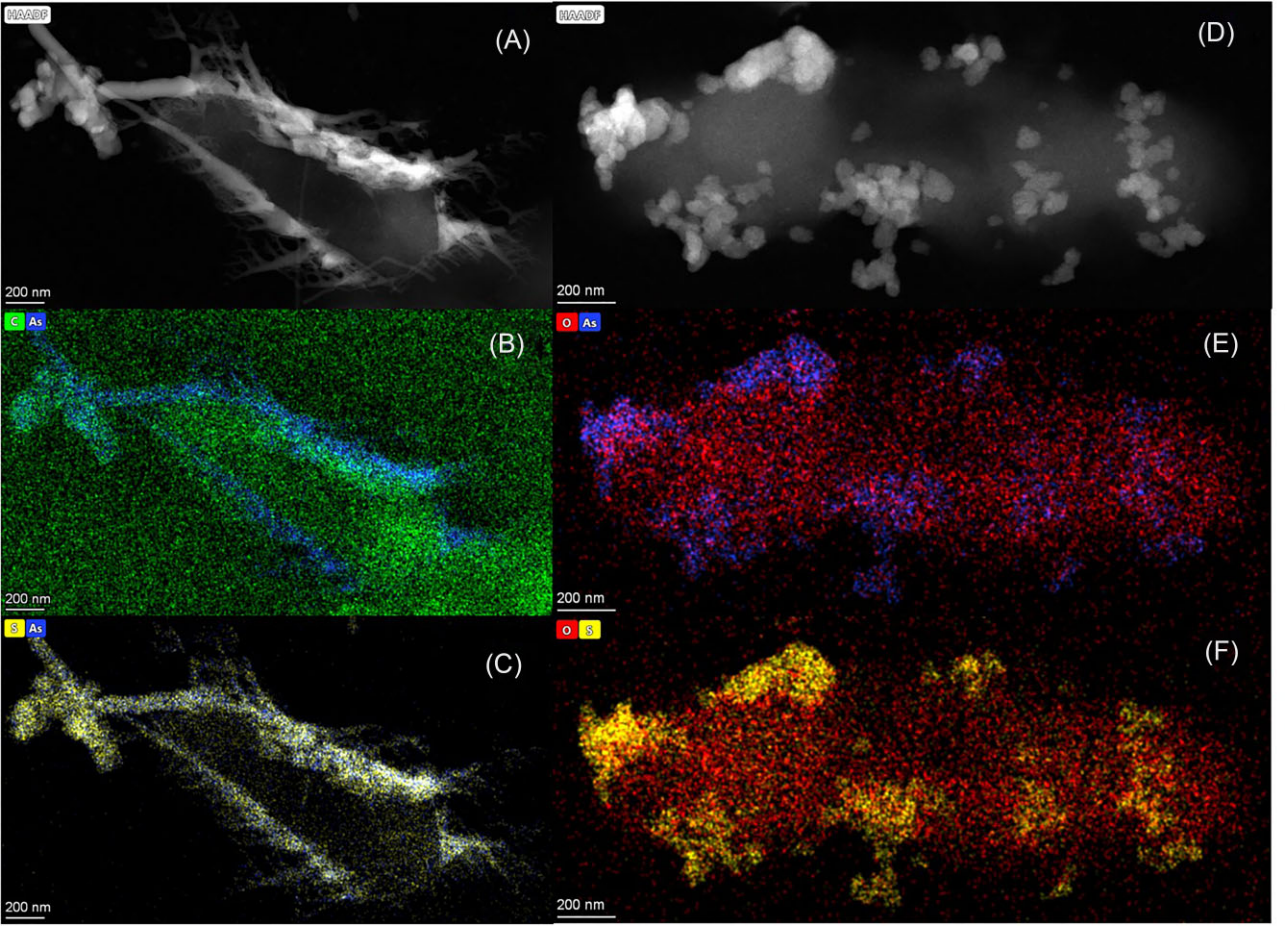
Biogenic AsS produced by *Shewanella* sp. O23S. Micrographs with the corresponding EDS elemental maps obtained from bacterial cells and biomineralization products: (A) Scanning transmission electron microscopy (STEM mode) of nanorod (AsS) structures; (B) Arsenic elemental mapping; (C) Sulfur elemental mapping; (D) Scanning transmission electron microscopy (STEM mode) of granular (As_2_S_3_) structures; (E) Arsenic elemental mapping; (F) Sulfur elemental mapping (Staicu et al. 2022a).

*Shewanella* sp. O23S can also respire arsenate (the *arr* operon is located on the pSheB plasmid) (Uhrynowski et al. 2019). When respiring arsenate to arsenite and degrading cysteine to H_2_S, the strain was shown to produce extracellular AsS biominerals of different morphologies: nanorod AsS (realgar) and granular (orpiment) As_2_S_3_ (Fig. 7).

Similar to biogenic Se^0^, AsS biominerals produced by this strain are amorphous, and the solutions exhibit a negative surface charge (-20 to -30 mV). Interestingly, when Se oxyanions and arsenate are mixed in the same system, the strain produces AsS and Se^0^ minerals in parallel (Staicu et al. 2022a). This suggests that the strain may utilize different biochemical pathways to transform As and Se. Indeed, the combination of the two elements results in both inhibitory and stimulatory outcomes in terms of reduction kinetics, depending on the oxyanion couple (arsenate-selenite and arsenate-selenate, respectively). It may be that *Shewanella* sp. O23S employs a multi-substrate respiratory enzyme. However, this does not exclude the presence of detoxification reactions in conjunction with the respiratory process. The strain possesses the detoxification operon (*ars*) on the same plasmid that harbors the *arr* operon (Uhrynowski et al. 2019), and it is widely established that selenium detoxification occurs through a number of strategies (e.g. glutathione reductase).

## Perspectives

The microbial formation of iron minerals, in particular by Fe(II)-oxidizing microorganisms but also by Fe(III)-reducers, has been studied intensively in the last two to three decades and the geochemical parameters controlling the identity and properties of iron biominerals are well understood. However, the potential for applying iron biominerals in bioremediation (removing pollutants such as certain toxic metals), for urban mining and biomining (harvesting precious resources such as phosphates and rare earth elements), as catalysts in biotechnological processes (e.g. biofuel production), and for energy/electron storage (biogeobatteries) has been underexplored so far and should be the focus of future studies.

Research on iron sulfide biomineralization has advanced considerably in the past few years. Nonetheless, intriguing questions remain, particularly regarding how microorganisms regulate the formation of extracellular greigite and pyrite framboids. Future cultivation studies that incorporate mixed microbial communities, fluctuating redox conditions, and natural sediment components (e.g. clays, calcite, quartz) may help to answer these questions.

Regarding S^0^, an open question remains to determine how S^0^-biomineralizing microbes control the properties of their biominerals, which differ significantly from their abiotic counterparts in terms of size, morphology, surface charge, or crystal structure. While experiments in abiotic (Cosmidis et al. 2019) and microbial systems (Cron et al. 2019) have revealed a strong influence of organics on S^0^ mineralogical properties, the specific biomolecules deployed by microbes to control their biomineralization products remain to be identified. A deeper understanding of these molecular controls would unlock the possibility to engineer S^0^ biominerals for different technological applications. S^0^ can, for instance, be applied in water treatment, where it can feed autotrophic denitrification. In addition, S^0^ reduction can be associated with organic matter remineralization or metal pollutant remediation, applications for which S^0^ properties such as bioavailability, small particle size, and hydrophilicity are critical (Zhang et al. 2021). There is also growing interest for using microbially derived components in battery technologies (Li et al. 2024), and it has been suggested that microbial S^0^ biominerals are interesting candidates to be used as cathode materials in high-capacity Li-S batteries, provided that we can gain better control of their yield and properties.

An open question related to biogenic AsS is whether the two observed morphologies (granules and wires/rods) are produced at the same time or if one morphology evolves from the other as a function of incubation time (e.g. AsS evolving from As_2_S_3_). The size of biogenic Se^0^ is puzzling, requiring clarification with regard to its intracellular formation and its potential export to the extracellular milieu without affecting the bacterial cell. In general, the intra- or extracellular localization of the minerals can pose various challenges to cellular integrity and viability. A major research topic pertains to the existence of biological functions in the genetically unregulated production of microbial biominerals—a fertile domain in need of future exploration (Cosmidis and Benzerara 2022).
